# Mental health in dance: A scoping review

**DOI:** 10.3389/fpsyg.2023.1090645

**Published:** 2023-02-23

**Authors:** Michelle Schachtler Dwarika, Heidi Marian Haraldsen

**Affiliations:** ^1^Sport, Exercise and Rehabilitation Sciences, Oslo National Academy of the Arts, University of Birmingham, Birmingham, United Kingdom; ^2^Dance Department, Oslo National Academy of the Arts, Oslo, Norway

**Keywords:** mental health, dance, ballet, dancers, dance education, stressors, mental process

## Abstract

Research in dance psychology and mental health is rapidly growing. Yet, evidence in the field can seem dispersed due to few existing meta overviews that outline research in dance related to mental health. Therefore, the aim of this scoping review is to strengthen future dance research by gathering and contextualizing existing findings on mental health in dance. Following the PRISMA guidelines and protocols, 115 studies were included in the review. Overall, the data analysis shows a predominant adoption of quantitative research but a lack of applied interventions of preventive and reactive procedures in mental health. Similarly, there is a tendency to study pre-professional dancers, whereas research into professional dancers, especially aged 30–60 is underrepresented. Dance genres have been unevenly investigated, with classical ballet being the most researched, whereas different dance styles and freelance employment are in dire need of in-depth investigation. Conceptualizing mental health as a dynamic state, the thematic analysis identified three main categories: *stressors, mental processes,* and *outcomes.* These factors appear to be in a complex interaction. Overall, the existing literature gives indications of components essential to understanding dancers’ mental health but has several blind spots and shortcomings. Therefore, a lot of in-depth understanding and research is still needed to fully grasp the dynamic complexity of mental health in dance.

## 1. Introduction

Research in dance psychology and mental health is rapidly growing. Yet, evidence in the field can seem dispersed due to few existing meta-overviews collecting and outlining research in dance and mental health. As dance science is a relatively new, vibrant, and evolving field, a scoping review of dance and mental health could strengthen future research by gathering and contextualizing existing findings (Moher et al., 2015). Therefore, we aimed to (a) examine how research is conducted in dance and mental health, (b) identify the scope of available evidence in the field of dance and mental health, and (c) identify factors that appear to represent and influence mental health in dance. In what follows, we begin by conceptualizing the scope and some crucial terms essential to the depicted data.

### 1.1. Mental health

In 2004, the World Health Organization redefined mental health as «a state of well-being in which the individual realizes his or her own abilities, can cope with the normal stresses of life, can work productively and fruitfully, and is able to make a contribution to his or her community» (World Health Organization, 2022). This marked a much-welcomed shift from seeing mental health not just as the absence of mental illness but encompassing mental well-being and thriving. However, it has been argued that this conceptualization is far from flawless (Galderisi et al., 2015). Scholars have argued that regarding well-being as a state of purely positive affect might be difficult to reconcile with certain complex circumstances we encounter in life (Keyes, 2002). People with good mental health might, for example, experience fluctuations of emotions such as fear, anger and sadness, or they can thrive in one environment or area of life and struggle in another (Galderisi et al., 2015; Henriksen et al., 2020). Also, different life events and transitions, such as change of schools or workplace, marriage and adolescent crises require that aspects of our mental health need to be reorganized, re-oriented or re-balanced (Galderisi et al., 2015). Therefore, it has been suggested to view mental health as a complete, dynamic state that encapsulates a broad spectrum of both the presence (flourishing) and absence (languishing) of mental health and the presence and absence of mental illness (Keyes, 2002; Küttel and Larsen, 2020). It further acknowledges that the strategies designed to reduce distressing symptoms might not be the same as those designed to enhance thriving or flourishing (Keyes, 2002; Küttel and Larsen, 2020). These mental strategies entail certain resources, such as the ability to relate to others, demonstrate psychological flexibility and cope with diverse stressors (Lazarus and Folkman, 1984; Galderisi et al., 2015). Additionally, this conceptualization of mental health also acknowledges that there exists a mutual relationship between the individual and its environment. An individual is influenced by the environment (e.g., amount and type of stressors) he or she is embedded in, and the environment is, in turn, affected by the persons in it (Keyes, 2002; Galderisi et al., 2015). Thus, it is not only the absence of psychological flexibility, relatedness or the ability to cope with diverse stressors, but also diverse and complex interactions between an individual and its environment that can result in mental health issues (Galderisi et al., 2015).

Recent headlines in the media remind us that the topic of mental health in dance seems of high relevance. Several European ballet institutions have currently been accused of sexual, physical and mental abuse of their dancers (Henley, 2019; Münch, 2019; Gregoris et al., 2022). In these accounts, young ballet students describe how they have been body shamed, humiliated or sexually harassed over several years. As a consequence, many of them are suffering from Post-traumatic stress disorder, depression or anxiety (Gregoris et al., 2022). Mental health in dance is, in other words, a pressuring matter. Therefore, this review examines existing literature on mental health in dance and discusses and promotes future research and attention in this area. Factors that might underpin this endeavor are *stressors*, *mental processes* and *mental health outcomes.*

#### 1.1.1. Stressors

An individual’s mental health resources are likely to be tested by *stressors* at different moments in their life (Fletcher and Sarkar, 2016). Such stressors can be defined as «environmental demands encountered by an individual» (Sarkar and Fletcher, 2014, p. 8; Mellalieu et al., 2006, p. 359) and are usually more modest disruptions to our everyday lives than major catastrophes (Sarkar and Fletcher, 2014). They are multifactorial and experienced on personal, cultural, and environmental levels. In this respect, sports research has investigated stressors in relation to their different states and recovery processes, or categorized them as competitive, organizational or personal (Kellmann, 2010; Sarkar and Fletcher, 2014). One dance-specific study has further identified physical stresses related to dance training, such as a high physical workload, and requirements concerning technical skill and choreographic demands; as well as psychosocial stressors related to the environment, such as managing finances and obligations, interrelations, and major life events (Blevins et al., 2020). Yet, a clear picture of the range and relevance of dance specific stressors do not exist to date.

#### 1.1.2. Mental processes

Mental health entails that individuals are affected by various factors such as context, situation and stressors but also encapsulates how they respond to and deal with these impacts and experiences (Lazarus and Folkman, 1984). Mental processes are therefore vital mechanisms that are often comprised of many steps towards a mental health outcome.

Sports research confirms that these processes are not linear but complex mechanisms that are comprised of many factors interacting with each other (Williams and Andersen, 1998; Fletcher and Sarkar, 2016). *Personal qualities* and the *environment* are essential overarching groups of resources in these mental processes. They can act in either protective or debilitative directions and increase or decrease mental health outcomes, respectively. They also represent and comprise several of the components identified to restore or strengthen mental health, such as the ability to relate to others, demonstrate psychological flexibility, and cope with challenging life events (Galderisi et al., 2015). Given the importance of these factors, it is vital to identify and map out an overview over these components to better grasp and address these complex mental processes in dance.

Personal qualities can be described as psychological factors that either protect or negatively influence individuals and must be distinguished from psychological skills (Fletcher and Sarkar, 2012, 2016). Personality, or personality characteristics, is a more stable, yet flexible, multilayered personal quality consisting of dispositional traits, characteristic adaptations, and self-narrative identities that “contribute to an individual’s distinctive patterns of feeling, thinking, and behaving” (Fletcher and Sarkar, 2016, p. 5). Psychological skills, on the other hand, are more adaptable cognitive and affective techniques and processes that are used to enhance and optimize an individual’s functioning or mental readiness in encountering stressors (Fletcher and Sarkar, 2016). A study in an academic setting, for example, has shown that students who become aware of the possibility of enhancing their personal qualities by training in psychological skills seem to enhance their flexibility and ability to cope with adversity (Yeager and Dweck, 2012). Therefore, an individual can train to acquire certain psychological skills that will enhance or improve their personality traits that thus act as protective factors against challenging life events.

Individuals are in a complex interaction with their environment. Consequently, their mental health is greatly influenced by different factors embedded in this climate. These can range from social and cultural circumstances or occurrences, such as auditions, transitions, injuries, deselection, and defeat (Fletcher and Sarkar, 2016), to stakeholders wielding power that influence the mental state of others. Therefore, environments can either be protective by nourishing a person’s mental health, or debilitative, by jeopardizing the balance of an individual’s mental health components and thus causing mental health challenges and disorders (Henriksen et al., 2020).

#### 1.1.3. Mental health outcomes

Mental processes can lead to either positive or negative *mental health outcomes*. Positive outcomes indicate the presence of mental health (flourishing; Keyes, 2002). In this case, the individual has enough personal resources to be protected from, adapt to, withstand, or swiftly rebound from an encounter with a stressor to avoid a permanent decrease in one’s mental health (Fletcher and Sarkar, 2016; Keyes, 2002). Consequently, this can lead to, increased task engagement and optimal performance. Negative outcomes can imply that stressors have exceeded available resources, leading to that the individual moves on a spectrum between the absence of mental health (languishing) and the presence of mental illness (Keyes, 2002). This can result in either mental health challenges, like distress, loneliness and exhaustion or mental illness as for example, depression, self-harm, and/or substance abuse (Howells and Fletcher, 2015). Yet, it is important to acknowledge the dynamic state of mental health and that individuals can thrive in one area of life and struggle in another (Henriksen et al., 2020). That means that individuals might sometimes succumb to a stressor but still experience states of mental well-being or that they benefit from the psychological and behavioral changes induced by this experience (Collins and MacNamara, 2012; Fletcher and Sarkar, 2016). Therefore, a *negative outcome* is not a permanent sentence of doom. Rather, it can lead to growth required for re-evaluation and reflection, and stimulate learning (Galderisi et al., 2015). To date, several studies in dance research have highlighted prevailing negative outcomes such as eating disorders, fatigue and trauma following injury occurrence (Schluger, 2010; Dantas et al., 2018; Kenny et al., 2019; van Winden et al., 2020). Yet, there exists, to our knowledge, no overview over positive and negative outcomes in dance research and little insight into which of these are prominent or obscure.

### 1.2. Research questions

Based on the relevant indications presented so far, this scoping review formulated the following research questions:

RQ1: What types of research designs, methodologies, publication sources, and populations are conducted in the research on dance and mental health?
RQ2: What are the identified stressors and mental health outcomes faced by Western theatre dance students, teachers, and professional dancers?
RQ3: Which factors appear to influence the dance students’, teachers’, and dancers’ mental health outcomes?

## 2. Methods

### 2.1. Context and population

To address the research questions, we created a protocol (Moher et al., 2015) in line with the purpose of a scoping review, that was to determine the scope or coverage of a body of literature on a given topic, how research has been conducted, and present an overview over its focus and existing literature (Munn et al., 2018). In contrast, a systematic review aims to identify and retrieve concrete evidence relevant to a particular question, establish the quality of the relevant evidence, and address uncertainty or variation in practice that may be occurring (Munn et al., 2018). Hence, we developed eligibility criteria framing the population, context, and concepts for the initial search phase. As the number of studies focusing on dance is still limited, this scoping review also included grey literature. Consequently, included studies were (a) peer-reviewed original research, or literature reviews, or systematic reviews, or master and PhD theses from 1980 to present. These studies were written in (b) Nordic or English language and (c) included samples of dance teachers, dance students and professional dancers age 13 and older (d) in the context of Western theatre dance (e.g., ballet, jazz, contemporary) and (e) the studies had to address mental health processes and outcomes according to the studies’ stated conceptualization.

### 2.2. Search strategy and procedure

The PRISMA guidelines were used during the screening and analysis process (see Table 1; Arya et al., 2021). The systematic search process consisted of several phases: initial search screening, main search screening, and supplemental manual search screening (Page et al., 2021). Librarians in a higher arts education institution assisted with conducting the initial and main screening procedures.

**Table 1:** Database research.

Database platform	Database	Number of results
EBSCOhost	International Bibliography of Theatre and Dance	1,065
	Education source	635
	ERIC	318
	Academic Search Ultimate	617
	SPORTDiscus	580
	Medline	214
Ovid	PSYCInfo	139
BIBSYS	Oria	325
–	(DUO)	(56)

Based on the protocol and its eligibility criteria, key terms, both in English and Norwegian, were formulated for the search string. The latter was then fed into each database according to the respective parameters. Search terms were tested individually and in different combinations to ensure viability within the search string. During this phase, a decision of removing conceptual words in the search string defining mental health concepts was taken, due to test searches indicating that concepts were limiting the scope, thus risking to narrow search outcomes. Instead, the authors decided to evaluate concepts manually during the first screening of the abstracts. Consequently, the search screened for population and context only. This resulted in the final search string in English that consisted of these terms and combinations: (“dance student*” OR dancer* OR “dance teacher*” OR “dance leader*” OR “ballet student*” OR “ballet teacher*” OR “ballet lead-er*”) AND (“western theatre dance” OR “dance education” OR “dance pedagog*” OR “classical ballet” OR “jazz dance” OR “contemporary dance” OR conservato* OR “talent identification” OR “development in dance” OR “aesthetic learning” OR apprentice). These terms and combinations were searched in relevant databases (see Table 1). The pre-determined limitations for each search within the databases were “apply equivalent subject” and “peer reviewed only.”

**Figure 1:**
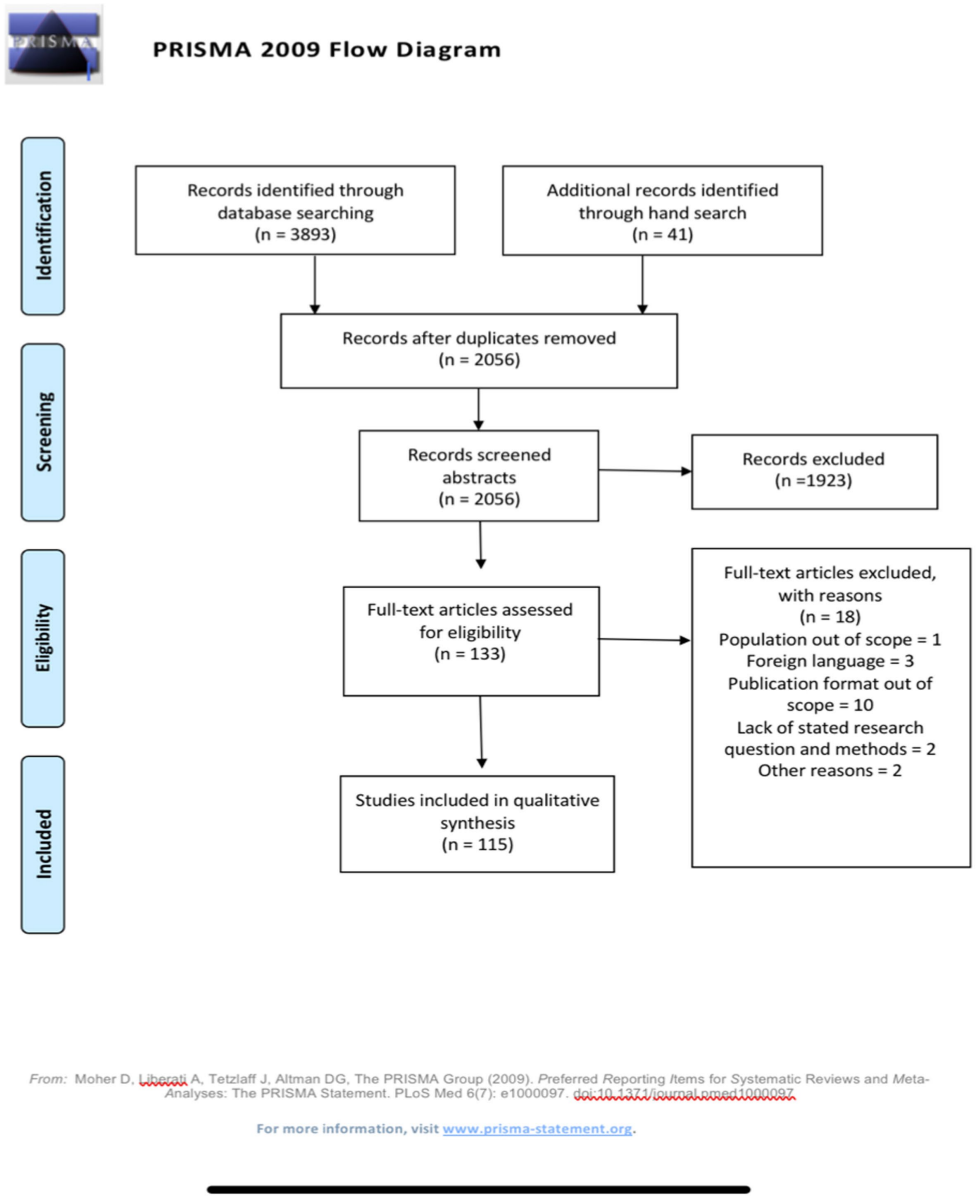
The flow chart of the screening process of identified and included studies.

Final searches were conducted on 16 February, 2021, which resulted in 3,893 retrieved articles prior to the removal of duplicates in Endnote (*N* = 1,865; Figure 1). Additional manual searches in the Norwegian thesis database, journals, and Google Scholar were undertaken to identify articles and theses that either were published recently (2020 to 2021) or were not identified by the initial search. Eventually, 2028 articles were uploaded to Rayyan, a free web-based app (Ouzzani et al., 2016), which the authors used as a screening tool to expedite the initial screening of abstracts and titles. A blind function in Rayyan enabled to execute a first screening on the abstract level, and labeling identified articles separately. After the first screening, a third party removed the blind function, and the authors then reviewed and discussed discrepancies in the excluded and included articles, rereading the abstracts and, if necessary, the articles in full text for further evaluation. Thereafter, the first author reviewed all articles in full text, taking the first steps toward data analysis and categorization. This resulted in the exclusion of further articles due to either (a) population out of scope, (b) foreign language, (c) publication format out of scope, (d) lack of stated research question and method section in the article and (e) other reasons, such as limited availability, which made up a new total of 115 included studies, as displayed in the flowchart (see Figure 1 for the flowchart and Table 1 for the overview over the included studies).

### 2.3. Data analyses and categorization

To extract data from the included studies, we created table sheets for (a) methodological categorization, (b) population and context information, (c) publishing sources, and (d) major foci of the studies. The first author filled in the different tables.

### 2.4. Thematic analysis and synthesis

We chose a qualitative and thematic approach (Booth et al., 2016) for presenting and synthesizing the results of this scoping review due to the wide range of research designs and thematic scope (Booth et al., 2016; Gough et al., 2017). In the thematic analysis, we (1) took a within-case approach, which entailed summarizing the main findings of all studies identified, (2) identified and developed descriptive themes and categories across the included studies, (3) summarized and developed overarching main findings within topics from a between-case approach (i.e., categories), and (4) meta-analyzed the findings to answer the research questions. The findings were categorized in accordance with an understanding of mental health as a complete and dynamic state (Keyes, 2002; Lazarus and Folkman, 1984). That means that individuals move on a broad spectrum between the presence (flourishing) or absence of mental health (languishing), and the presence or absence of mental illness (Keyes, 2002). The authors met regularly during the analysis process to discuss, and peer debrief the emerging meanings and results (Barber and Walczak, 2009).

## 3. Results

### 3.1. Scope of research design and methodology

Research question 1 of this scoping review examined, how research is conducted in dance and mental health. Data analysis from the categorization of the included studies showed that there were 52 quantitative studies, 39 qualitative investigations, and 25 mixed study designs (Supplementary Table S2). Of these, 32 were longitudinal, and 83 were cross-sectional studies.

**Figure 2:**
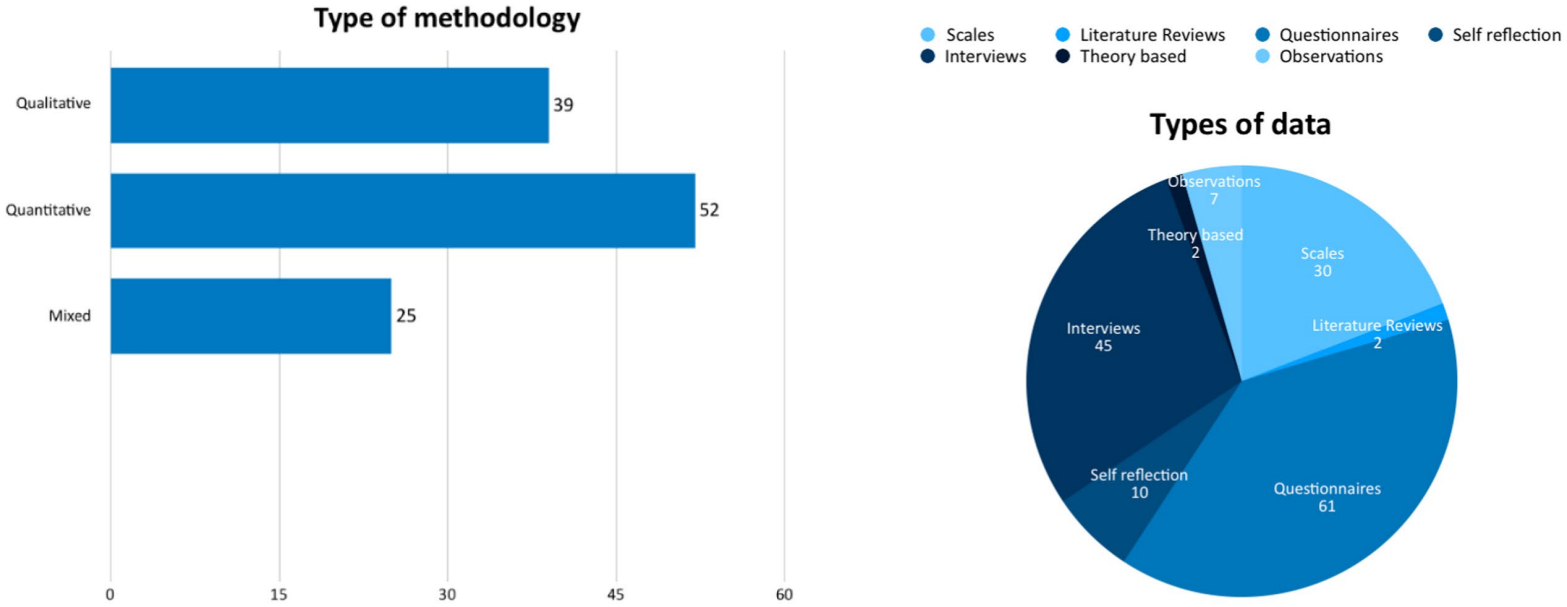
Distribution of research design and methodology.

The distribution of the methodology and type of data (Figure 2) shows that the majority of the studies in this review were non-experimental and descriptive (155), followed by experimental (5) and reviews (2). Regarding types of data, questionnaires, interviews, and scales dominated. Scales were describing all kinds of tests or pre-determined screening tools that were used to test variables such as eating disorders.

**Figure 3:**
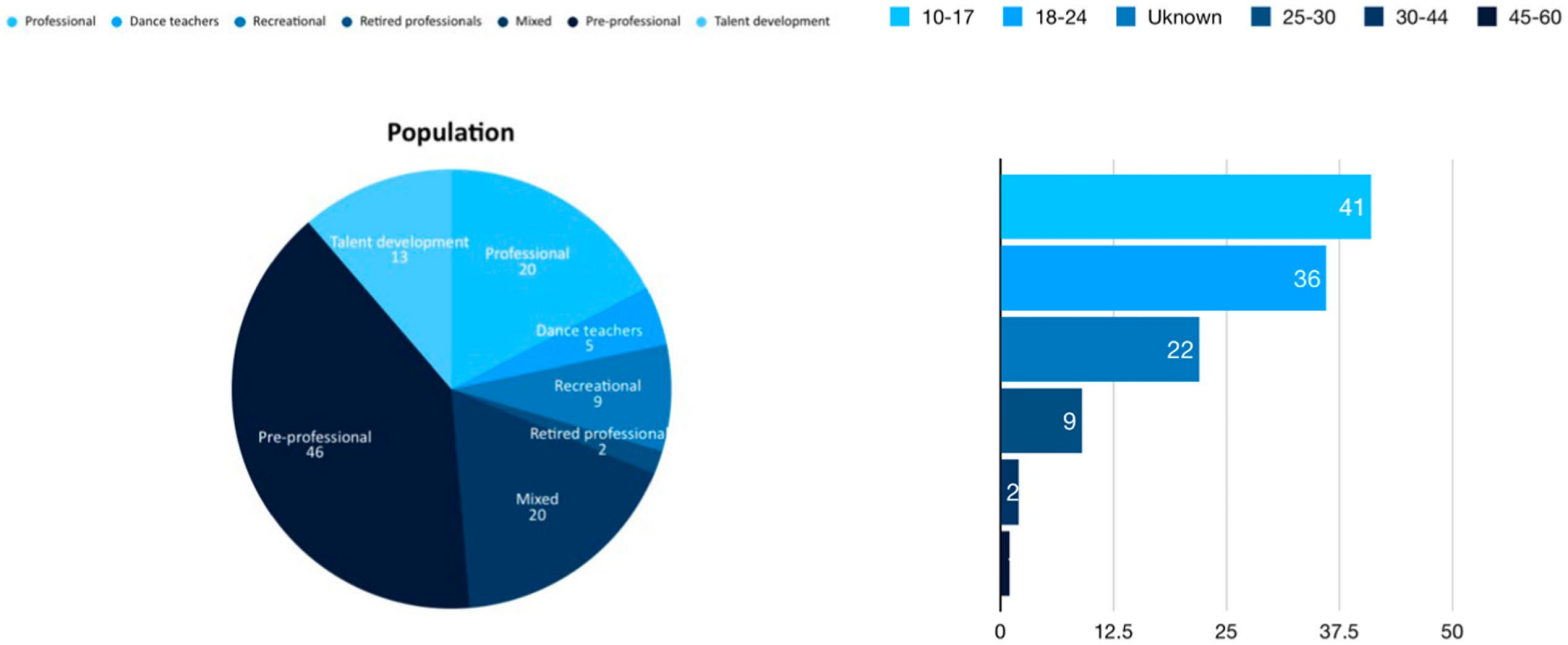
Overview of researched population and age-range.

The population distribution (see Figure 3) indicated that pre-professional dance students in high school or higher education programs (46 studies), professional dancers (20), and mixed population (20 studies, i.e., different combinations of pre-professional and professional dancers or recreational dancers) were the most researched population. This may be due to the easy access to participants and relevance to the examined topics. Dancers in specified talent development programs were investigated in 13 studies. Dance teachers (5) and retired professionals (2) seemed generally underrepresented in research. This is also reflected in the age distribution, with dancers aged 10–17 dominating the analyzed studies (45), followed by participants aged 18–24 (36) and 25–30 (9). Dancers aged 30–44 were researched in two studies, whereas those aged 45–60 were represented in only one study.

Further, dance genres were unequally represented, with classical ballet being the most researched (76), followed by contemporary/modern dance (32), jazz (5), and hip hop (3). However, these numbers also combine studies examining several dance genres. In these cases, the examined dance genres were counted separately.

Examining the representation of different nationalities and diversity in the research, the Western countries positioned in West Europe and North America dominated the analyzed studies. The review also revealed that a few dance journals dominated and contained the majority of publications on general mental health investigations (see Supplementary Table S1).

### 3.2. Thematic scope and synthesis

The thematic analysis identified numerous stressors and diverse resources needed to meet and cope with the stressors, as well as aspects of both positive and negative mental health outcomes. This resulted in the following three main themes: (1) stressors, (2) mental processes and (3) mental health outcomes. Each will now be presented in turn.

#### 3.2.1. Stressors

Several studies in this review describe how individuals encountered various environmental demands, also called stressors. They are organized as *situational*, *interpersonal, or cultural.* Each group of stressors is presented in turn in the following paragraphs (see Table 2).

**Table 2:** Journals with more than one publication.

Publishing Source	Studies	Ratings
Journal of Dance Education	49	**0.7** CiteScore
		**0.295** SJR
Research in Dance Education	27	**1.5** CiteScore
		**0.310** SJR
Journal of Dance Medicine and Science	17	**0.7** CiteScore
		**0.288** SJR
Dissertation Abstract International	6	
Theatre, Dance and Performance Training	6	**0.4** CiteScore
		**0.279** SJR
NTNU Open	5	
UBIRA e-theses	5	
Collections of Canada	4	
Dance Research Journal	4	**0.49** CiteScore
		**0.19** SJR
Medical Journal of Performing Arts	3	**1.5** CiteScore
		**0.307** SJR
Frontiers in Psychology	3	**4.232** CiteScore
		**0.947** SJR
Journal of Dance and Somatics	2	**0.24** CiteScore
		**0.109** SJR
High Ability Studies	2	**3.2** CiteScore
		**0.334** SJR
Sex roles	2	**4.24** CiteScore
		**1.309** SJR

*Situational stressors* (20 studies) in general include career uncertainty, time management issues, limited economic means and injuries. Only a few articles in this category describe how limited financial means and career uncertainty represent issues negatively affecting dancers’ development and potential future (Sanchez et al., 2013; Lopez, 2019). On the other hand, injuries are a much more discussed topic (Liederbach and Compagno, 2001; Kenny et al., 2019; van Winden et al., 2020; Pentith et al., 2021). Injuries negatively affect dancers in many ways, hindering training and performance, and thus, hampering the learning and development process (Macchi and Crossman, 1996). Also, dealing with an injury is mentally tough, and dancers’ mental states appear to influence how effectively these are coped with (Macchi and Crossman, 1996; Mainwaring et al., 2001; Kenny et al., 2019). Therefore, several studies stress the importance of therapists, sport psychologists, medical professionals, and teachers to provide a holistic approach to injury management (Macchi and Crossman, 1996; Mainwaring et al., 2001; Pollitt and Hutt, 2021).

*Interpersonal stressors (26 studies)* are related to asymmetric power exerted by authority figures, perceived pressure and expectations from others, and body image pressure from peers and teachers. Several studies describe that these factors place tacit and rigid demands on the dancers (Benn and Walters, 2001; van Staden et al., 2009; Dantas et al., 2018; Haraldsen et al., 2021a). Power exerted by authority figures, such as teachers and choreographers, seem to influence dancers’ outward agreements with a set cultural system (Benn and Walters, 2001; Parker, 2011; Pickard, 2013; Dantas et al., 2018). Peers are often part of this system and influence, alongside the teachers, dancers’ body image, eating attitudes and overall ideals (Table 3).

**Table 3:** Overview over themes and their meanings.

Stressor	The mental process	Mental health outcomes
**Situational stressors (20)** Career uncertainty Time management issues Limited economic means Injury **Interpersonal stressors (26)** Asymmetric power relations Perceived pressure and expectations from others Body image pressure from peers and teachers **Cultural Stressors (42)** Cultural hegemony Physical ideals Narrow-minded identity ideals Cult-like behavior Traditional gender roles Hierarchical and top-down organizations	**Facilitative process** **Protective factors:** *Protective Personal qualities* Proactive personality (37) Relatedness (21) Confidence (13) Harmonious passion (7) Optimism (4) *Facilitative environment* (23) Mastery-climate Task-oriented learning culture Autonomy supportive teachers Focus on self-development Psychologically safe and caring Progressive and student-centered teaching style **Debilitative process** **Debilitative factors:** *Debilitative personal qualities* Perfectionism (16) Obsessiveness (14) Ego-orientation (10) *Unrelenting environment* (49) Expectations of conforming to cultural ideals Performance pressure Ego-involving climate Pressure to fit into the dance world Authoritarian teaching style Competition and comparison	**Positive outcome (53)** *Presence of mental health (Flourishing)* Increased life quality Positive emotional states Increased confidence Proactive self and career management Increased self efficacy Critical thinking ability and autonomy Holistic and diverse identity Work / life balance **Negative outcome (76)** *Absence of mental health (Languishing)* Lack of relatedness, loneliness Fatigue and exhaustion Trauma following injury occurrence Distress Decreased self worth Feelings of inadequacy and failure Debilitated life quality *Presence of mental illness* Eating disorders Depression Burnout (Performance) Anxiety

*Cultural stressors* (42 studies) describe factors inherent in dance culture, such as cultural hegemony, set physical ideals, narrow minded identity ideals, cult-like behavior expectations, traditional gender roles, and hierarchical and top-down organizations. Ballet is described as an authoritarian, hierarchical, cult-like power achievement culture where dancers accept abuse and unreasonable behavior in a state of «silent conformity» (Benn and Walters, 2001; Parker, 2011). Part of this culture are set, physical ideals which affect dancers to strive for thinness to attain a ballet physique or ideal dancers’ body (Benn and Walters, 2001; Dryburgh and Fortin, 2010; Pickard, 2013; Mitchell et al., 2020). Other studies describe narrow minded identity ideals inherent in the dance culture. That means that dancers are expected to possess and display certain personality characteristics, such as being docile, humble, hard working, dedicated, mentally tough and persistent (van Staden et al., 2009; Parker, 2011; Aujla et al., 2014, 2015; Haraldsen et al., 2019, 2020). Finally, male adolescent dancers appear seven times more likely than the general public to be bullied, teased or harassed – regardless of their sexual orientation (Risner, 2014). Negativity, stereotypes, bias, and harassment are accepted as commonplace and thus expected, negotiated, and endured (Risner, 2014). In particular, male ballet dancers report engaging in a system that is characterized by gendered rules in both technique and performance, highly stigmatized as effeminate and gender codified (Haltom and Worthen, 2014).

#### 3.2.2. Mental processes

The processes of handling stressors described in the studies are broadly categorized either as *facilitative* or *debilitative*. Facilitative processes comprise proactive and more robust personal qualities on the one side and protective aspects of the dance environment on the other. Together, these factors appear to either restore or strengthen mental processes and thus act as protective factors in coping mechanisms. Debilitative processes comprise dysfunctional personal qualities and unrelenting features in the dance environments that seemingly jeopardize or imbalance mental health processes. The results of how each of these were identified in the data will now be presented in turn.

##### 3.2.2.1. Facilitative process

###### 3.2.2.1.1. Protective personal qualities

According to the data analysis, five personal qualities were associated with individuals who withstand stressors: *positive personality, confidence, relatedness, harmonious passion, and optimism*.

*Positive personality* (37 studies) describes dancers that are striving for self-actualization, self-assessment, self-efficacy, self-management, autonomy, self-development, flexibility, and versatility. Several studies suggested that these factors aid dancers to form a holistic identity or buffer stressors they encounter (van Staden et al., 2009; Klockare et al., 2011; Mitchell et al., 2017; Blevins et al., 2020; de Las Heras Fernández et al., 2020). However, according to the included studies, these qualities are deemed in need of development, nurturing, and strengthening, not only in dance but in future dance research in general.

The data analysis identified several studies examining the role of *confidence* (13 studies) in the dance literature. The majority of these articles describe dancers’ lack of confidence in relation to body image, career transitions, and gender identity. Yet, they also present suggestions on how to increase confidence. This entails enhancing autonomous, creative explorations, developing skills beyond the dance world and nurturing relationships (Green, 1999; Dearborne et al., 2006; Watson et al., 2012; Aujla et al., 2014; Haltom and Worthen, 2014). Similar to previous factors, it is recommended that confidence and its related themes should be further examined in future research.

*Relatedness (21 studies)* describes the ability to establish and maintain social relationships with friends, family, peers, teachers, and organizations. Apart from acknowledging these relationships as essential, dance research also repeatedly points to the stress-buffering effect of the perception and experience of social support (Li, 2011; Walker et al., 2012; Aujla et al., 2014; Risner, 2014; Reis et al., 2019).

*Harmonious passion* (7 studies) is considered a flexible and autonomous approach to involvement in dance, in which the individual participates of his/her own volition, and the activity does not dominate his/her identity (Aujla et al., 2015). This entails striking a dance-life balance that offers room for the building and maintenance of friendships and nurturing other interests/hobbies outside of the dance realm. Our results showed that harmonious passion strengthened support systems, contributed to the shaping of more flexible identities and increased motivation and adherence, which potentially eases career transitions (Aujla et al., 2015).

*Optimism* (4) is explicitly mentioned in the data of a few studies, either describing the lack of optimism dancers have or suggesting measures to increase optimism in this population (Macchi and Crossman, 1996; Kveton-Bohnert, 2017; Wenn et al., 2018; Senning, 2020). Generally, these articles identified optimism as important for mental health, but also in need of further investigation.

###### 3.2.2.1.2. Facilitative environment

Generally, *facilitative environments* (23) are mastery-oriented motivational climates that are task-oriented, nurture dancers’ autonomy and self-development and are perceived as psychologically safe (Benn and Walters, 2001; Mainwaring et al., 2001; Critien and Ollis, 2006; Quested and Duda, 2009; Haraldsen et al., 2020). Several studies highlighted that dancers regulated by self-determined motivation appeared more robust and engaged in a healthier, harmonious way in their development (Quested and Duda, 2009; Hancox et al., 2017; Haraldsen et al., 2020, 2021a). Teachers play an essential role in both the creation and in the perception of these climates (Carr and Wyon, 2003; Hancox et al., 2017; Wenn et al., 2018). In this respect, studies show that an autonomy supportive and student-centered teaching style nurture high motivational quality, dancers’ self-determined motivation, and create more harmonious development paths in dance (Quested and Duda, 2009; Haraldsen et al., 2019, 2020, 2021a).

##### 3.2.2.2. Debilitative process

###### 3.2.2.2.1. Debilitative personal qualities

Data analysis identified three main personal qualities that appeared to nurture stressors that jeopardize or imbalance mental processes: *perfectionism, obsessiveness and ego-orientation*.

*Perfectionism* (16 studies) is related to fear of failure, self-critique, overly evaluative processes, and linking self-worth to achievements (Stornæs et al., 2019). Maladaptive perfectionism can entail a large perceived discrepancy between performance and personal standards, that individuals doubt themselves and avoid negative consequences (Van Staden et al., 2009). This might result in conditional self-worth, risk of over-training or the use of avoidance strategies (Haraldsen et al., 2021a). Therefore, perfectionistic tendencies might contribute to color the perception of and the way dancers cope with the dance environment (Liederbach and Compagno, 2001; Nordin-Bates et al., 2014; Kenny et al., 2019; van Winden et al., 2020; Pentith et al., 2021).

*Obsessiveness* (14 studies) describes the way dancers exhibit compulsive striving, which can be described as a perceived need for progress and internalized pressures for achievement at the expense of their social and emotional needs and development (van Staden et al., 2009). These strivings appear to be nurtured by the dance cultural ideals, pre-determined identities and expected behaviors, such as dedication and mental toughness, and for male dancers, they may also include debunking stereotypes and enduring homophobia, heterosexism bias, and harassment (van Staden et al., 2009; Polasek and Roper, 2011; Radell et al., 2014; Haraldsen et al., 2021a). Thus, compulsive striving often entails forms of self-objectification that can lead to lack of self-awareness, self-alienation, and to dancers isolating themselves from their social life (van Staden et al., 2009). Similarly, evidence showed that these ideals and ingrained values can lead to obsessive passion, which is described as a rigid persistence to participate in dance, often resulting in dancing taking up disproportionate importance in an individual’s identity and leaving little space for other interests, decreasing their overall life quality (Aujla et al., 2015; Cahalan et al., 2019).

*Ego orientation* (10 studies) is a form of external motivation that depicts a tendency to focus on outperforming others and demonstrating superior ability (Carr and Wyon, 2003). To ego-oriented individuals, high effort implies low ability whereas low effort indicates high ability. This view is negatively affecting their efforts and learning opportunities. It is also linked to avoidance strategies concealing lack of competence and fear of failure (Carr and Wyon, 2003). A strong sense of competitiveness and comparison seems to enhance ego-orientation. Studies point to environmental factors, such as teaching style, motivational climate and cultural ideals as strong contributing factors to ego-orientation (van Staden et al., 2009; Pickard, 2013; Haraldsen et al., 2020).

###### 3.2.2.2.2. Unrelenting environment

Overall the majority of studies describe the dance environment as *unrelenting* (49 studies). This is a climate that has tacit or explicit expectations to conform to ideals, exerts pressure to perform and to fit the mould dictated by the dance world. Gatekeepers, such as teachers, choreographers, and artistic directors, have often been successful performers themselves and, thus, wield a lot of power and authority in this climate and dance culture in general (van Staden et al., 2009; Dryburgh and Fortin, 2010; Haraldsen et al., 2020).

Teachers in an *unrelenting environment* often adopt an authoritarian teaching style and tend to compare their students and thus enhance peer-competition (Benn and Walters, 2001; Quested and Duda, 2009; van Staden et al., 2009; Haraldsen et al., 2019, 2020). Consequently, revealing incompetence or disloyalty, or disappointing these stakeholders represents a risk to hamper dancers’ social position, career opportunities, or their chances for further development (Haraldsen et al., 2020). Overall, characteristics of an unrelenting environment within dance showed little care for dancers’ mental health, unhealthy competition, and the tacit or explicit expectation to conform to ideals and expectations were the most important features.

#### 3.2.3. Mental health outcomes

**Figure 4:**
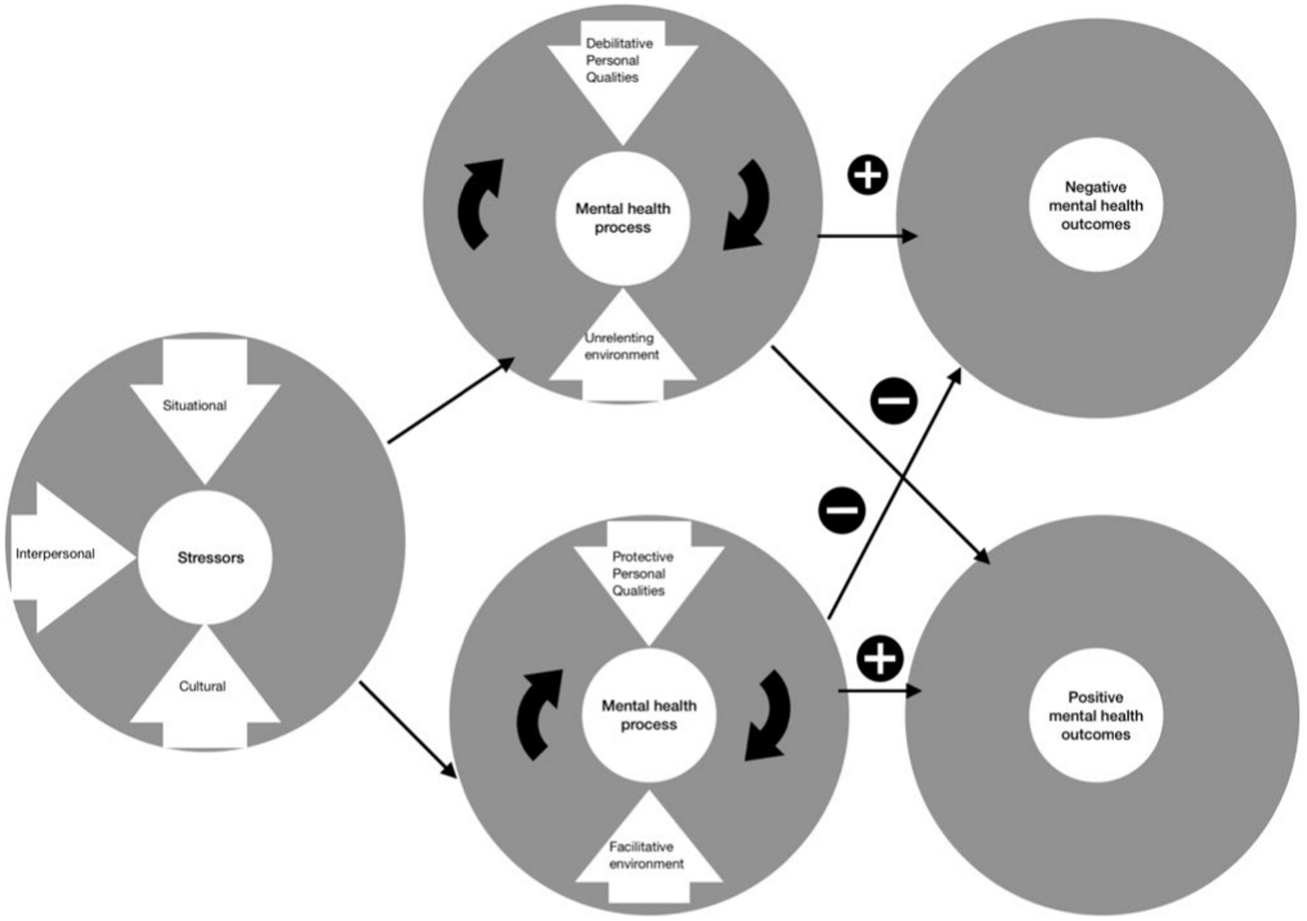
Overview over a mental health process.

Depending on the number of perceived stressors, as well as the features and interaction of the personal qualities and environment at hand, diverse mental health outcomes seemingly increased or decreased (see Figure 4).

##### 3.2.3.1. Positive outcomes (53)

The results identified a range of positive mental health outcomes in dance. Life quality, confidence and self efficacy and positive emotional states, proactive self and career management, and the nurturing of holistic identities are among the most reported outcomes of flourishing mental health characteristics. However, these results were mostly identified in studies that also reported the absence of mental health. Positive outcomes were often presented as possible, future effects rather than being identified as prevalent. Moreover, dancers seem generally to have low confidence and possess little knowledge related to how to manage their careers and increase their quality of life (Hoffer, 1981; van Staden et al., 2009; Haraldsen et al., 2021a). Yet, studies indicated that the dancers’ life quality could increase if measures, such as psychological skills training and psycho-education, were systematically applied in their education and professional life (Redding and Quested, 2006; Solomon et al., 2002; Diaz et al., 2008; van Staden et al., 2009; Klockare et al., 2011; Carattini, 2020; Kim et al., 2022). So far, positive outcome evidence appeared dispersed and lacked coherence, which made it difficult to detect an overall prevalence.

##### 3.2.3.2. Negative outcomes (61)

Generally, the absence of mental health (languishing) led to mental health challenges, such as distress, loneliness, stress and tiredness. Mental illnesses like, anxiety, and eating disorders are among the most described negative outcomes (Slater and Tiggemann, 2002; van Staden et al., 2009; Risner, 2014; van Winden et al., 2020). Moreover, there are indications that ballet dancers are at a higher risk of developing eating disorders than contemporary dancers (Benn and Walters, 2001; Schluger, 2010; Nordin-Bates et al., 2011; Dantas et al., 2018). Also, perfectionism seems a common predictor variable for eating disorders, performance anxiety, and burnout in both male and female dancers (Nordin-Bates et al., 2011, 2014; Haraldsen et al., 2021a,b). However, while some studies compared dancers to other population (Adame et al., 1991; Archinard and Scherer, 1995; Slater and Tiggemann, 2002; Pollatou et al., 2010; Spadafora, 2010; Kosmidou et al., 2017; Walter and Yanko, 2018), not enough coherent evidence was identified to secure an overall prevalence in this review.

## 4. Discussion

In this section, we present an overall meta-reflection and discussion of the findings presented in the results section. We start by discussing the research designs, methodology, and quality, and continue with the thematic analysis of the main findings within the scope and its subcategories. We conclude with a discussion of limitations, and future research.

### 4.1. Research design and methodology

The first research question focused on what type of research design, methodology, and population could be detected in the included studies. Our analysis showed that quantitative methods were dominating in the current literature, specifically, descriptive observational survey studies based on self-reported questionnaire data. The qualitative studies, which also were primarily descriptive in nature, were mostly based on interview studies. Hence, the diversity and sophistication of research designs and methodology was low. This affected the type of research questions to be asked, likely conclusions that could be drawn and the impact on the overall quality of the research (Teddlie and Tashakkori, 2011). This is shown in, for example, by a lack of building on previous studies identifying stressors in the literature. Even though the studies have pointed to many and varied problems that affect dancers’ mental health, central aspects in mental processes (e.g., moderator and mediator variables), such as appraisals, responses, or resilience, are not properly explored, identified, or investigated. Moreover, only a few of the studies were intervention studies that tested out the effect of strategies to reduce or encounter previously identified stressors. Yet, methodological research literature highlights the importance of intervention studies (Welsh et al., 2023, p. 18). Intervention studies especially might be beneficial to test theories and put them into practice. Further, there are shortcomings concerning the conceptualization of mental health. There are tendencies to explore mental health in a general manner, with no given definition of the conceptualization in the context of the given study. For example, the concept of well-being is often used as an expression of mental health, underpinned by a variety of measurement (i.e., vitality, positive affect, self-determination) but also by an array of labels and constructs (i.e., psychological well-being, mental well-being, thriving or flourishing; van Staden et al., 2009; Aujla and Farrer, 2015; van Winden et al., 2020). Except for eating disorders, specific in-depth investigations of mental issues such as depression, anxiety or emotional distress are not yet conducted. Hence, this review points to many potential future research topics within Western dance to be examined. One important starting point is that mental health and its dynamic nature needs to be defined and conceptualized in dance.

The analysis of the range of population showed a preoccupation with pre-professional dancers and higher education students from Western countries, with ballet being by far the most researched dance genre. This may be due to ballet and its characteristics presenting easily measurable variables, which further highlights that in-depth research into other dance genres and freelance dancers is long overdue. From a critical viewpoint one might claim that the evidence concerning dance and mental health represent a story of young Western ballet dance students, and not a representation of the broad field of artistic dance itself.

### 4.2. Thematic scope and trends

To address the last two research questions that identified stressors, influential factors and mental health outcomes in dance literature, we undertook a thematic analysis that identified three main themes: *stressors, mental processes and mental health outcomes.* Each of these is discussed in turn in the following sections.

#### 4.2.1. Stressors

The results identified a range of stressors comprised of *situational*, *interpersonal*, and *cultural*. In the context of Western dance theatre, cultural stressors appear to be the most influential, followed by interpersonal stressors. The extent to which of these stressors manifested themselves varied among the dance genres dissected in the data. This became especially apparent when analyzing the reviews’ *cultural stressors.* This revealed that genres such as jazz are more concerned about body image, gender identities, and commercialization of the body than contemporary and modern dance, which seem more progressive in the cultivation and reflection of positive body image (Heiland et al., 2008; Pollatou et al., 2010; Alexias and Dimitropoulou, 2011; Swami and Harris, 2012)*. Interpersonal stressors* identified many aspects of pressures. It appeared, for example, that not only teachers but also peers exerted pressure on dancers’ body image and perceived expectations (Dantas et al., 2018). Classical ballet especially seemed irrevocably connected to deep-running traditions and a fostering of *cultural and interpersonal stressors*, which various participants in several studies reported to be “part of the deal” and as an aspect that is tacitly expected, accepted, and cultivated (Benn and Walters, 2001; Risner, 2014; Cahalan et al., 2019; Haraldsen et al., 2019). Other challenges and factors, such as *situational stressors*, are underrepresented across the scoping review and could therefore not provide a much-needed understanding of factors such as dancers’ financial means, support systems, and environments (Sanchez et al., 2013). Several studies describe injuries as a central stressor that jeopardizes dancers’ mental health (Macchi and Crossman, 1996; Backlund and Wallén, 2016; Cahalan et al., 2019; Haraldsen et al., 2021a). Although injuries are characterized by physical challenges, the findings highlight the importance of interconnecting dancers’ physical and mental needs when dealing with an injury. Furthermore, dancers in all dance genres generally appear to possess or receive little knowledge about how to attend to injuries, rest, deal with mental health issues, and build a life outside of the dance environment (Macchi and Crossman, 1996; Aujla et al., 2014; van Winden et al., 2020). Overall, there is a need to further the understanding of the interaction between physical and mental health in dance and test out the effect of mental health education as part of dance education and dance teacher training.

#### 4.2.2. Mental processes

Findings in the literature suggest that dancers’ mental health is influenced by both *protective* and *debilitative* factors. However, tendencies in the identified studies seem to indicate, that dancers are either languishing *or* are showing signs of thriving and flourishing. This appearance of a little nuanced picture of dancers’ mental health seem to be underpinned by several factors. First, that there is little rigorous conceptualization of mental health in dance; second, that there is a prevalence of quantitative studies in the field that might not capture the more complex, in-depth aspects and dynamic states of mental health; and third, that research on mental health is generally not practically applied in interventions or other experimental designs.

The evidence relating to *protective personal qualities* revealed that buffering attributes, such as confidence, were presented as suggestions for further research rather than practically applied and explored (Kveton-Bohnert, 2017; Blevins et al., 2020; Carattini, 2020; Kim et al., 2022). Therefore, *protective personal qualities* should be seen as useful suggestions and indications for further research, rather than established evidence in the dance context. *Facilitative environment,* on the other hand, are underpinned by more in-depth endeavors that uncover which elements are likely to contribute to dancers thriving in their environment. The majority of these investigations focus on motivational climates and imply that awareness of the three basic psychological needs from self-determination theory and motivational quality in general can make a positive difference in dancers’ lives (Quested and Duda, 2009; Hancox et al., 2017; Haraldsen et al., 2020). However, while motivational quality and climates appear to be researched to some degree in dance, there is still limited knowledge about dancers’ motivational states in different dance genres. Also, to date, other important aspects, such as psychological safety, have been little explored and require further investigation.

In general, there was a prevalence in studies describing debilitative aspects inherent in an *unrelenting environment* (such as performance pressure and ego-involving climate), that, in turn, were linked to certain debilitative personal factors such as perfectionism or obsessiveness. Even though research on perfectionism conceptualizes the term and examines it contextually (Nordin-Bates et al., 2011, 2014; Stornæs et al., 2019), both ego-orientation and obsessiveness lack conceptualizations and contextual approaches. We know, for example, little about the dimensions of obsessiveness and how aspects, such as compulsive striving, look like in different dance genres. So far, these *debilitative personal qualities* seem inextricably linked to an *unrelenting environment* that appears to enable peers and teachers to influence dancers’ body image, eating attitudes, and overall ideals, which in turn hamper dancers’ development and well-being (Critien and Ollis, 2006; Lacaille et al., 2007; Harper, 2012; Stanway et al., 2020; Haraldsen et al., 2021a). Several of the studies offer valuable advice how to address and change debilitative aspects of the particular cultural features of the dance environment (Bonbright, 1995; Mainwaring et al., 2001; Batur et al., 2003; van Staden et al., 2009; Schluger, 2010). However, to date almost none of these plans have been put into action and tested for viability and effect.

Overall, the findings in this scoping review indicate that we possess little knowledge about the mental processes in dance. For instance, no study, to date, has investigated concepts of resilience in dance. However, sport psychology studies have conducted investigations into coping strategies, stress and recovery as well as resilience processes. They highlight that gaining knowledge about whether an individual appraises a stressor as a threat or challenge is decisive in the process of how the stressor is perceived and dealt with (Fletcher and Sarkar, 2016). Future research needs to address and reflect whether different components such as appraisals and responses might further the understanding of mental health processes in dance. Furthermore, research should capture the dynamic nature of dancers’ mental health processes. These should investigate whether, and in which way dancers’ *protective* and *debilitative personal* qualities coexist and how they interact and adapt during different time periods.

#### 4.2.3. Mental health outcomes

This review captured both *positive* and *negative mental health outcomes.* Findings identified the presence of mental health by means of increased life quality, positive emotional states, establishing a work/life balance, and using psychological skills to increase confidence, self and career management. (van Staden et al., 2009; Nordin-Bates et al., 2011; Kveton-Bohnert, 2017; Hopper et al., 2020; Kim et al., 2022). The most frequent indications for the absence of mental health were stress, distress and tiredness related to burnout. The presence of mental illness was indicated by negative outcomes such as anxiety and eating disorders. Of these, eating disorders and psychological trauma following injury occurrence especially, seem to be deeply influenced by dance environments’ ingrained power culture and ballet aesthetics, as well as by factors of significance such as mirrors (Liederbach and Compagno, 2001; Kenny et al., 2019; van Winden et al., 2020; Pentith et al., 2021). Thus, the strong indications for both the absence of mental health and the presence of mental illness, point to a most warranted change in the dance world to address these issues.

Prevalence has been difficult to identify, but would be needed in order to gain a more precise overview of prevalence in mental health outcomes. Studies examining, for example, how dancers compare to the general public or other athletes, would aid the understanding of mental health and the debilitative impact of mental health issues in dance. Overall, positive and negative outcomes appear to exist side by side, despite endeavors to study them as separate entities. That means that dance research needs to look at the holistic picture, modeling and testing the sum of stressors, resources available in the process and the composition of the individual’s health situation, in order to understand these outcomes (see Figure 4). However, to date dance research still lacks replication studies and more experimental designs that test and verify mental health components. It is important to consider how these could compose a more holistic picture of dancers’ mental health.

### 4.3. Limitations

This scoping review has several limitations. First, the nature of a scoping review is to present trends and an overview of the scope of existing literature, and thus cannot present a detailed and in-depth analysis of the findings. This leaves research question 3 challenging to answer in concrete terms. Second, the inclusion of grey studies, such as master thesis, might be a limitation given the quality of such included studies. However, as dance research is a rather young research field, master theses have, so far, played an important part in contributing to the overall research evidence, and thus, excluding these could have resulted in an incomplete picture of the existing literature. Third, the disperse evidence and the general lack of replication studies challenges the demonstration of prevalence in a variety of mental issues and disorders. Fourth, no structured and detailed quality assessment has been undertaken. This might be a general weakness of scoping reviews that could have strengthened the reader’s assessment of the included studies. However, an assessment of the quality of the journals with the most included studies, gives a general overall quality assessment. Fifth, how these findings are presented, which definition of mental health has been chosen to underpin this endeavor, and what research gaps that are detected, are interlinked with the perceptions, opinions, and background of the researchers, despite their striving to avoid bias and practice reflexivity. This means that the authors also are reflected in the synthesis and meaning making of this review. Since few reviews of this kind exist in dance literature, it has been our endeavor to present a review that offers connection points from which readers can make sense of existing literature on mental health in dance. Therefore, the authors hope that this scoping review can, despite its limitations, be seen as a valuable puzzle piece to a much bigger jigsaw.

### 4.4. Concluding remarks

In this scoping review, we have tried to summarize and synthesize what appears to be dispersed studies on the matter of mental health and its determinants in dance. In general, dance students and dancers are exposed to a unique range of stressors that might potentially increase their vulnerability to the absence of mental health or mental illness. On the other hand, in the process towards their flourishing mental health state, both personal and environmental qualities seem to be important contributors in the total equation. As several studies in this review have shown, a deeper and applied understanding of the interaction between *stressors, mental health processes* and its *outcomes* are essential to gain insight into and grasp the dynamic nature of mental health. Hence, this scoping review suggests that mental health in dance should be conceptualized as a complete and dynamic state. However, the synthesized picture of mental health in dance is far from complete and seems still quite anecdotical in nature-revealing topics uncovered, populations left out, and too little diversity and rigor in the methodological approaches. Insofar, the current review has contributed to advance the scope of knowledge about mental health in dance and intends to initiate a more informed discussion about how we can better understand, conceptualize, measure, and support dancers’ mental health.

## Author’s note

Research in dance psychology and mental health is rapidly growing. Yet, evidence in the field can seem dispersed due to few existing meta overviews that outline research in dance related to mental health and, especially, facilitative, or debilitative processes associated with mental health outcomes. Therefore, the aim of this scoping review is to present an overview of the state of the art and to strengthen future dance research by gathering and contextualizing existing findings on mental health in dance. This effort has revealed that factors such as personal qualities, stressors, appraisals, responses, and the impacts of the environment are useful indicators for understanding facilitative or debilitative mental health processes. In turn, these are associated with positive or negative mental health outcomes but also point to gaps that need to be filled. Thus, this scoping review both synthezises disperse evidence in the field but is also aims to present a foundation for future research in mental health in dance.

## Author contributions

All authors listed have made a substantial, direct, and intellectual contribution to the work and approved it for publication.

## Funding

This scoping review was funded by the Oslo National Academy of the Arts’ Dance Department.

## Conflict of interest

The authors declare that the research was conducted in the absence of any commercial or financial relationships that could be construed as a potential conflict of interest.

## Publisher’s note

All claims expressed in this article are solely those of the authors and do not necessarily represent those of their affiliated organizations, or those of the publisher, the editors and the reviewers. Any product that may be evaluated in this article, or claim that may be made by its manufacturer, is not guaranteed or endorsed by the publisher.
