# Supplementary material for: Mental health in dance: A scoping review
Source: Front Psychol. 2023 Feb 23;14:1090645. doi: 10.3389/fpsyg.2023.1090645 (PMC10035338; doi:10.3389/fpsyg.2023.1090645)
Supplement: Supplementary file 1 [file Table_1.docx]

*Supplementary Table 1: Overview over included studies*

|  |
| --- |
| 1. Adame, D. D., Radell, S. A., Johnson, T. C., & Cole, S. P. (1991). Physical fitness, body image, and locus of control in college women dancers and nondancers. *Perceptual and motor skills*, *72*(1), 91-95. 2. Alexias, G., & Dimitropoulou, E. (2011). The body as a tool: Professional classical ballet dancers’ embodiment. *Research in Dance Education*, *12*(2), 87-104. 3. Aujla, I. J., Nordin-Bates, S. M., Redding, E., & Jobbins, V. (2014). Developing talent among young dancers: Findings from the UK Centres for Advanced Training. *Theatre, dance and performance training*, *5*(1), 15-30. 4. Aujla, I. J., Nordin-Bates, S., & Redding, E. (2014). A qualitative investigation of commitment to dance: findings from the UK Centres for Advanced Training. *Research in dance education*, *15*(2), 138-160. 5. Aujla, I. J., Nordin-Bates, S. M., & Redding, E. (2015). Multidisciplinary predictors of adherence to contemporary dance training: findings from the UK Centres for Advanced Training. *Journal of sports sciences*, *33*(15), 1564-1573. 6. Archinard, M., & Scherer, U. (1995). Training in classical dance in childhood and adolescence: What influence on bulimia nervosa in adult patients?. *European Eating Disorders Review*, *3*(2), 111-116. 7. Backlund, H., & Wallén, F. (2016). Pre-professionella balettdansares upplevelser och erfarenheter av dansrelaterade skador. 8. Batur, S., Kabakci, E., & Gulol, C. (2003). Maladaptive eating attitudes of elite and amateur Turkish dancers: are they at risk?. *Eating and Weight Disorders-Studies on Anorexia, Bulimia and Obesity*, *8*(4), 263-267. 9. Benn, T., & Walters, D. (2001). Between Scylla and Charybdis. Nutritional education versus body culture and the ballet aesthetic: The effects on the lives of female dancers. *Research in dance education*, *2*(2), 139-154. 10. Bennett, D. (2009). Careers in dance: Beyond performance to the real world of work. *Journal of Dance Education*, *9*(1), 27-34. 11. Blevins, P., Erskine, S., Hopper, L., & Moyle, G. (2020). Finding Your Balance: An Investigation of Recovery–Stress Balance in Vocational Dance Training. *Journal of Dance Education*, *20*(1), 12-22. 12. Blevins, P., Erskine, S., Moyle, G., & Hopper, L. (2020). Student and teacher attitudes towards overtraining and recovery in vocational dance training. *Theatre, Dance and Performance Training*, *11*(1), 5-24. 13. Bonbright, J. M. (1995). *Discriminating physiological, behavioral and psychological characteristics in eating disorders: Clinical and psychometric comparisons between clinically diagnosed patients, ballet dancers and controls* (Doctoral dissertation, Temple University). 14. Bottamini, G. L. (2000). *Ballet teachers: A source of perceived weight loss pressure in female ballet students*. University of Ottawa (Canada). 15. Cahalan, R., Comber, L., Gaire, D., Quin, E., Redding, E., Ni Bhriain, O., & O'sullivan, K. (2019). Biopsychosocial characteristics of contemporary and Irish University-level student dancers a pilot study. *Journal of Dance Medicine & Science*, *23*(2), 63-71. 16. Carattini, C. M. (2020). *Psychological skills in ballet training: An approach to pedagogy for the fulfilment of student potential* (Doctoral dissertation, Queensland University of Technology). 17. Carr, S., & Wyon, M. (2003). The impact of motivational climate on dance students' achievement goals, trait anxiety, and perfectionism. *Journal of Dance Medicine & Science*, *7*(4), 105-114. 18. Chua, J. (2014). Dance talent development across the lifespan: A review of current research. Research in Dance Education, 15(1), 23-53. 19. Chua, J. (2015). The role of social support in dance talent development. Journal for the Education of the Gifted, 38(2), 169-195. 20. Chua, J. (2017). The influences of an exemplary ballet teacher on students’ motivation:‘The Finnish Way’. Research in Dance Education, 18(1), 3-22. 21. Clements, L., & Nordin-Bates, S. M. (2020). Inspired or Inhibited? Choreographers’ Views on How Classical Ballet Training Shaped Their Creativity. *Journal of Dance Education*, 1-12. 22. Critien, N., & Ollis, S. (2006). Multiple engagement of self in the development of talent in professional dancers. *Research in Dance Education*, *7*(2), 179-200. 23. Dantas, A. G., Alonso, D. A., Sánchez-Miguel, P. A., & del Río Sánchez, C. (2018). Factors dancers associate with their body dissatisfaction. *Body image*, *25*, 40-47. 24. Dearborn, K., Harring, K., Young, C., & O'Rourke, E. (2006). Mirror and phrase difficulty influence dancer attention and body satisfaction. *Journal of Dance Education*, *6*(4), 116-123. 25. de las Heras Fernández, R., Espada Mateos, M., Carrascal Dominguez, S., & Garcia Coll, V. (2020). Evaluation and analysis of emotional intelligence, mood and coping strategies in two Spanish dance companies. *Research in Dance Education*, *21*(3), 231-244. 26. Diaz, H., Chatfield, S. J., & Cox, J. (2008). Cultivating Presence in Movement: Student Refections on the Practice of Feldenkrais Awareness Through Movement® in Dance Training. *Journal of Dance Education*, *8*(3), 79-93. 27. Demelius, Y. (2003). Steps of a dance production: the working lives of professionals at a dance company (Doctoral dissertation, Concordia University). 28. Downs, S. V. C. (2013). *Coping and injury in a professional ballet company: an investigation of stressors, appraisal, coping processes and injury in professional ballet dancers* (Doctoral dissertation, University of Birmingham). 29. Dryburgh A. & Fortin S. (2010): Weighing in on surveillance: perception of the impact of surveillance on female ballet dancers’ health, *Research in Dance Education, Volume 1, number 2* 30. Fietze, I., Strauch, J., Holzhausen, M., Glos, M., Theobald, C., Lehnkering, H., & Penzel, T. (2009). Sleep quality in professional ballet dancers. *Chronobiology international*, *26*(6), 1249-1262. 31. Flower, L. (2019). Spiritual experiences of post-performance career ballet dancers: a qualitative study of how peak performance spiritual lived experiences continued into and influenced later teaching lives. *Research in Dance Education*, *20*(2), 184-196 32. Gittens, C. Y. (2012). *A comparative study: Perfectionism in elite ballet dancers and artistic gymnasts* (Doctoral dissertation, University of Birmingham). 33. Green, J. 1999. Somatic authority and the myth of the ideal body in dance education. Dance Research Journal, 31(2): 80–100. 34. Green, J. 2003. Foucault and the training of docile bodies in dance education. Arts and Learning Research, 19(1): 99–126. 35. Gruzelier, J. H., Thompson, T., Redding, E., Brandt, R., & Steffert, T. (2014). Application of alpha/theta neurofeedback and heart rate variability training to young contemporary dancers: State anxiety and creativity. *International Journal of Psychophysiology*, *93*(1), 105-111. 36. Haltom, T. M., & Worthen, M. G. (2014). Male ballet dancers and their performances of heteromasculinity. *Journal of College Student Development*, *55*(8), 757-778. 37. Hamilton L.; Hamilton, W.G.; Warren, M.P.; Keller, K. and Molnar, M. (1997): Factors contributing to the attrition rate in elite ballet students. *Journal of Dance Medicine and Science, Volume 1, number 4.* 38. Hancox, J. E., Quested, E., Ntoumanis, N., & Duda, J. L. (2017). Teacher-created social environment, basic psychological needs, and dancers' affective states during class: A diary study. *Personality and Individual Differences*, *115*, 137-143. 39. Hancox, J. E. (2014). *Examination of the social-environmental and motivational processes operating in dance contexts: a self-determination theory approach* (Doctoral dissertation, University of Birmingham). 40. Haraldsen, H. M., Abrahamsen, F. E., Solstad, B. E., & Halvari, H. (2021). Narrative Tensions in Strained Junior Elite Performers’ Experiences of Becoming Elite Performers. Frontiers in Psychology, 12, 1767. 41. Haraldsen, H. M., Halvari, H., Solstad, B. E., Abrahamsen, F. E., & Nordin-Bates, S. M. (2019). The role of perfectionism and controlling conditions in Norwegian elite junior performers’ motivational processes. Frontiers in psychology, 10, 1366. 42. Haraldsen, H. M., Nordin-Bates, S. M., Abrahamsen, F. E., & Halvari, H. (2020). Thriving, Striving, or Just Surviving? TD Learning Conditions, Motivational Processes and Well-Being Among Norwegian Elite Performers in Music, Ballet, and Sport. Roeper Review, 42(2), 109-125. 43. Haraldsen, H. M., Solstad, B. E., Ivarsson, A., Halvari, H., & Abrahamsen, F. E. (2020). Change in basic need frustration in relation to perfectionism, anxiety, and performance in elite junior performers. Scandinavian journal of medicine & science in sports, 30(4), 754-765. 44. Harper, S. (2012). *A critical comparative study of career transition policy, practice and experiences for ballet company dancers and musical theatre independent dancers* (Doctoral dissertation, University of Birmingham). 45. Heiland, T. L., Murray, D. S., & Edley, P. P. (2008). Body image of dancers in Los Angeles: the cult of slenderness and media influence among dance students. *Research in Dance Education*, *9*(3), 257-275. 46. Hoffer, D. L. (1981). *The classical ballet dancer: a psycho-social analysis of the dance personality profile*. Nova University. 47. Hopper, L. S., Blevins, P., Erskine, S., Hendry, D., Hill, R., & Longbottom, R. (2020). Sustaining dancer wellbeing through independent professional dance careers. *Theatre, Dance and Performance Training*, *11*(4), 470-486. 48. Hrušová, D. (2015). Effect of dancing on subjective experiences and psychological state of dancers. 49. Karin, J., & Nordin-Bates, S. M. (2020). Enhancing Creativity and Managing Perfectionism in Dancers Through Implicit Learning and Sensori-Kinetic Imagery. *Journal of Dance Education*, *20*(1), 1-11. 50. Kartawidjaja, J. E., & Cordero, E. D. (2013). Fat talk and body dissatisfaction among college dancers. *Journal of Dance Education*, *13*(4), 122-129. 51. Kenny, S. J., Palacios-Derflingher, L., Shi, Q., Whittaker, J. L., & Emery, C. A. (2019). Association between previous injury and risk factors for future injury in preprofessional ballet and contemporary dancers. *Clinical journal of sport medicine*, *29*(3), 209-217. 52. Kerr, J. H., Fujiyama, H., Wilson, G. V., & Nakamori, K. (2006). The experience of a modern dance group: arousal, motivation, and self‐rated performance. *Research in Dance Education*, *7*(2), 125-140. 53. Kim, H., Tasker, S. L., & Shen, Y. (2020). How to persevere in a ballet performance career: exploring personal wisdom of retired professional ballet dancers. *Research in Dance Education*, 1-26. 54. Klockare, E., Gustafsson, H., & Nordin-Bates, S. M. (2011). An interpretative phenomenological analysis of how professional dance teachers implement psychological skills training in practice. *Research in dance education*, *12*(3), 277-293. 55. Kosmidou, E., Giannitsopoulou, E., & Moysidou, D. (2017). Social Physique Anxiety and pressure to be thin in adolescent ballet dancers, rhythmic gymnastics and swimming athletes. *Research in Dance Education*, *18*(1), 23-33. 56. Kushida, M. A. (2017). The Environment and Identity Processes: The Dancer Identity as a Case Study *(Doctoral dissertation, UC Riverside).* 57. Kveton-Bohnert, L. A. (2017). *The Voices of Classical Ballet Dancers: Alleviating Maladaptive Perfectionism through Resilience, Mindful Learning, and Self-Compassion* (Doctoral dissertation, Saybrook University). 58. Lacaille, N., Koestner, R., & Gaudreau, P. (2007). On the value of intrinsic rather than traditional achievement goals for performing artists: A short-term prospective study. *International Journal of Music Education*, *25*(3), 245-257. 59. Li, Z. (2011). Adolescent males in dance: A closer look at their journey. *Journal of Physical Education, Recreation & Dance*, *82*(7), 17-23. 60. Liederbach, M., & Compagno, J. M. (2001). Psychological aspects of fatigue-related injuries in dancers. *Journal of Dance Medicine & Science*, *5*(4), 116-120. 61. Lopez, B. (2019). Dance students at a two year college: making sense of their academic, cultural, and social world. *Research in Dance Education*, *20*(2), 174-183. 62. Macchi, R., & Crossman, J. (1996). After the fall: reflections of injured classical ballet dancers. *Journal of Sport Behavior*, *19*(3), 221. 63. Mainwaring, L., Krasnow, D., & Young, L. (2003). A teacher's guide to helping young dancers cope with psychological aspects of hip injuries. *Journal of dance Education*, *3*(2), 57-64. 64. May, J., Redding, E., Whatley, S., Łucznik, K., Clements, L., Weber, R., ... & Reed, S. (2020). Enhancing creativity by training metacognitive skills in mental imagery. *Thinking Skills and Creativity*, *38*, 100739. 65. Minton, S. C. (2001). Assessment of high school dance students' self-esteem. *Journal of Dance Education*, *1*(2), 63-73. 66. Mitchell, S. B., Haase, A. M., & Cumming, S. P. (2021). Of Grit and Grace: Negotiating Puberty, Surviving, and Succeeding in Professional Ballet. *Women in Sport and Physical Activity Journal*, *1*(aop), 1-12. 67. Mitchell, S. B., Haase, A. M., & Cumming, S. P. (2020). Experiences of delayed maturation in female vocational ballet students: An interpretative phenomenological analysis. *Journal of adolescence*, *80*, 233-241. 68. Mitchell, S. B., Haase, A. M., Cumming, S. P., & Malina, R. M. (2017). Understanding growth and maturation in the context of ballet: a biocultural approach. *Research in Dance Education*, *18*(3), 291-300. 69. Nieminen, P. (1998). Participation motives in relation to background and involvement variables among Finnish non-professional dancers. *Dance Research Journal*, *30*(2), 53-70. 70. Nieminen, P., Varstala, V., & Manninen, M. (2001). Goal orientation and perceived purposes of dance among Finnish dance students: A pilot study. Research in dance education, 2(2), 175-193. 71. Nordin-Bates, S. M. (2020). Striving for Perfection or for Creativity? A Dancer’s Dilemma. *Journal of dance education*, *20*(1), 23-34. 72. Nordin-Bates, S. M., Walker, I. J., & Redding, E. (2011). Correlates of disordered eating attitudes among male and female young talented dancers: Findings from the UK Centres for Advanced Training. *Eating disorders*, *19*(3), 211-233. 73. Nordin-Bates, S. M., Walker, I. J., Baker, J., Garner, J., Hardy, C., Irvine, S. & Blevins, P. (2011). Injury, imagery, and self-esteem in dance healthy minds in injured bodies?. *Journal of Dance Medicine & Science*, *15*(2), 76-85. 74. Nordin-Bates, S. M., Cumming, J., Aways, D., & Sharp, L. (2011). Imagining yourself dancing to perfection? Correlates of perfectionism among ballet and contemporary dancers. *Journal of Clinical Sport Psychology*, *5*(1), 58-76. 75. Nordin-Bates, S. M., Hill, A. P., Cumming, J., Aujla, I. J., & Redding, E. (2014). A longitudinal examination of the relationship between perfectionism and motivational climate in dance. *Journal of Sport and Exercise Psychology*, *36*(4), 382-391. 76. Parker, R. (2011). *An exploration of the identity issues faced by retiring male ballet dancers* (Doctoral dissertation, University of Birmingham). 77. Pentith, R., Moss, S., Lamb, K., & Edwards, C. (2020). Perfectionism Among Young Female Competitive Irish Dancers: Prevalence and Relationship with Injury Responses. 78. Peters, P. J. (2020). Grit for Dance Students: What, Why, and How. *Dance Education in Practice*, *6*(1), 7-12. 79. Petrides, K. V., Niven, L., & Mouskounti, T. (2006). The trait emotional intelligence of ballet dancers and musicians. *Psicothema*, *18*, 101-107. 80. Pickard, A. (2013). Ballet body belief: Perceptions of an ideal ballet body from young ballet dancers. *Research in Dance Education*, *14*(1), 3-19. 81. Pickard, A., & Bailey, R. (2009). Crystallising experiences among young elite dancers. *Sport, Education and Society*, *14*(2), 165-181. 82. Polasek, K. M., & Roper, E. A. (2011). Negotiating the gay male stereotype in ballet and modern dance. *Research in Dance Education*, *12*(2), 173-193. 83. Pollatou, E., Bakali, N., Theodorakis, Y., & Goudas, M. (2010). Body image in female professional and amateur dancers. *Research in Dance Education*, *11*(2), 131-137. 84. Pollitt, E. E., & Hutt, K. (2021). Viewing Injury in Dancers from a Psychological Perspective-A Literature Review. *Journal of Dance Medicine & Science*, *25*(2), 75-79. 85. Puddu, C. (1998). The relationship between stress and eating attitudes and behaviour in female dancers and field hockey players. 86. Quested, E., & Duda, J. L. (2009). Perceptions of the motivational climate, need satisfaction, and indices of well-and ill-being among hip hop dancers. *Journal of Dance Medicine & Science*, *13*(1), 10-19. 87. Radell, S. A., Adame, D. D., & Cole, S. P. (2004). The impact of mirrors on body image and classroom performance in female college ballet dancers. *Journal of Dance Medicine & Science*, *8*(2), 47-52. 88. Radell, S. A., Keneman, M. L., Adame, D. D., & Cole, S. P. (2014). My body and its reflection: a case study of eight dance students and the mirror in the ballet classroom. *Research in Dance Education*, *15*(2), 161-178. 89. Redding, E., & Quested, E. (2006). When Art Meets Science. *International Journal of Learning*, *13*(7). 90. Reis, N. M., Cardoso, A. A., de Araújo, C. R., Moratelli, J., Boing, L., Borgatto, A. F., & de Azevedo Guimarães, A. C. (2019). Quality of life is associated with fatigue among Brazilian professional dancers. *Motricidade*, *15*(1), 33-38. 91. Risner, D. (2002). Sexual orientation and male participation in dance education: Revisiting the open secret. Journal of Dance Education, 2(3), 84-92. 92. Risner, D. (2014). Bullying victimisation and social support of adolescent male dance students: an analysis of findings. *Research in Dance Education*, *15*(2), 179-201. 93. Rodrigues, F. R., e Cunha, M. P., Castanheira, F., Bal, P. M., & Jansen, P. G. (2020). Person-job fit across the work lifespan–The case of classical ballet dancers. *Journal of Vocational Behavior*, *118*, 103400. 94. Sanchez, E. N., Aujla, I. J., & Nordin-Bates, S. (2013). Cultural background variables in dance talent development: findings from the UK centres for advanced training. *Research in Dance Education*, *14*(3), 260-278. 95. Schluger, A. E. (2010). Disordered eating attitudes and behaviors in female college dance students: Comparison of modern dance and ballet dance majors. *North American Journal of Psychology*, *12*(1), 117-128. 96. Senning, C. B. (2020). *A Study on the Confluence of Resilience Skill Building and Contact Improvisation on Collegiate Dancers* (Doctoral dissertation, UC Irvine). 97. Skaardal, E. (2006). *Kroppen som middel og symbol: en kvalitativ studie av unge jenters bearbeidelse av prestasjoner og kroppsidealer i dansen* (Master's thesis). 98. Slater, A., & Tiggemann, M. (2002). A test of objectification theory in adolescent girls. *Sex Roles*, *46*(9), 343-349. 99. Solomon, R., Solomon, J., Micheli, L. J., Saunders, J. J., & Zurakowski, D. (2002). Using the test of attentional and interpersonal style (TAIS) for profiling and counseling dancers. *Journal of Dance Medicine & Science*, *6*(4), 119-127. 100. Spadafora, A. (2010). Predictors of eating pathology in adolescents: a comparison of ballet dancers and non-dancers. 101. Stanway, A. R., Fein, E. C., & Bordia, S. (2020). The moderating role of conscientiousness between psychological contract breach and loyal boosterism. *Research in Dance Education*, *21*(1), 82-97. 102. Stracciolini, A., Stein, C. J., Kinney, S., McCrystal, T., Pepin, M. J., & Meehan III, W. P. (2017). Associations between sedentary behaviors, sleep patterns, and BMI in young dancers attending a summer intensive dance training program. *Journal of Dance Medicine & Science*, *21*(3), 102-108. 103. Stornæs, A. V., Rosenvinge, J. H., Sundgot-Borgen, J., Pettersen, G., & Friborg, O. (2019). Profiles of perfectionism among adolescents attending specialized elite-and ordinary lower secondary schools: A Norwegian cross-sectional comparative study. *Frontiers in psychology*, *10*, 2039 104. Swami, V., & Harris, A. S. (2012). Dancing toward positive body image? Examining body-related constructs with ballet and contemporary dancers at different levels. *American Journal of Dance Therapy*, *34*(1), 39-52. 105. Tvedt, M. S. (2011). *Danseelevers målorientering og opplevelse av det motivasjonelle klima i relasjon til selvoppfatning og emosjonelle problem* (Master's thesis, University of Stavanger, Norway). 106. van Staden, A., Myburgh, C. P., & Poggenpoel, M. (2009). A psycho-educational model to enhance the self-development and mental health of classical dancers. *Journal of Dance Medicine & Science*, *13*(1), 20-28. 107. van Winden, D., van Rijn, R. M., Savelsbergh, G. J., Oudejans, R. R., & Stubbe, J. H. (2020). Limited Coping Skills, Young Age, and High BMI Are Risk Factors for Injuries in Contemporary Dance: A 1-Year Prospective Study. *Frontiers in Psychology*, *11*, 1452. 108. Van Zelst, L., Clabaugh, A., & Morling, B. (2004). Dancers' Body Esteem, Fitness Esteem, and Self-esteem in Three Contexts. *Journal of Dance Education*, *4*(2), 48-57. 109. Walker, I. J., Nordin-Bates, S. M., & Redding, E. (2012). A mixed methods investigation of dropout among talented young dancers: Findings from the UK Centres for Advanced Training. *Journal of Dance Medicine & Science*, *16*(2), 65-73. 110. Walker, I. J., Nordin-Bates, S. M., & Redding, E. (2011). Characteristics of talented dancers and age group differences: findings from the UK Centres for Advanced Training. High Ability Studies, 22(1), 43-60. 111. Walker, I. J., Nordin‐Bates, S. M., & Redding, E. (2010). Talent identification and development in dance: A review of the literature. Research in Dance Education, 11(3), 167-191. 112. Walter, O., & Yanko, S. (2018). New observations on the influence of dance on body image and development of eating disorders. *Research in Dance Education*, *19*(3), 240-251. 113. 1Wanke, E. M., Schmidt, M., Leslie-Spinks, J., Fischer, A., & Groneberg, D. A. (2015). Physical and mental workloads in professional dance teachers. *Medical problems of performing artists*, *30*(1), 54-60 114. Watson, D. E., Nordin-Bates, S. M., & Chappell, K. A. (2012). Facilitating and nurturing creativity in pre-vocational dancers: Findings from the UK Centres for Advanced Training. *Research in Dance Education*, *13*(2), 153-173. 115. Wenn, B., Mulholland, R., Timmons, W., & Zanker, Y. (2018). Towards a developing construct in dance education–exploring the relation of emotional intelligence to teacher’s sense of efficacy and teaching experience among dance education student teachers in the United Kingdom. *Research in Dance Education*, *19*(1), 14-38. |
